# Brain perfusion SPECT in the presurgical evaluation of epilepsy: is additional ictal SPECT required in case of high-confidence lateralization of the seizure onset zone by interictal SPECT and vice versa?

**DOI:** 10.1186/s13550-024-01149-8

**Published:** 2024-09-12

**Authors:** Kian Baradaran-Salimi, Amir Karimzadeh, Berthold Voges, Ivayla Apostolova, Thomas Sauvigny, Olga Simova, Michael Lanz, Susanne Klutmann, Stefan Stodieck, Philipp T. Meyer, Ralph Buchert

**Affiliations:** 1 Department of Diagnostic and Interventional Radiology and Nuclear Medicine, University Medical Center Hamburg-Eppendorf, Martinistr. 52, 20246 Hamburg, Germany; 2 Department of Neurology and Epileptology, Protestant Hospital Alsterdorf, Hamburg, Germany; 3 Department of Neurosurgery, University Medical Center Hamburg-Eppendorf, Hamburg, Germany; 4 Department of Nuclear Medicine, Medical Center - University of Freiburg, Faculty of Medicine, University of Freiburg, Freiburg, Germany

**Keywords:** Epilepsy, SPECT, Perfusion, Cerebral blood flow, Interictal, Ictal, SISCOM, Seizure onset zone, ^99m^Tc-HMPAO, ^99m^Tc-ECD

## Abstract

**Background:**

Ictal brain perfusion SPECT provides higher sensitivity for the identification of the epileptic seizure onset zone (SOZ) than interictal SPECT. However, ictal SPECT is demanding due to the unpredictable waiting period for the next seizure to allow for ictal tracer injection. Thus, starting with an interictal scan and skipping the ictal scan if the interictal scan provides a SOZ candidate with high confidence could be an efficient approach. The current study estimated the rate of high-confidence SOZ candidates and the false lateralization rate among them for interictal and ictal SPECT.

**Methods:**

177 patients (48% females, median age 38y, interquartile range 27–48y) with ictal and interictal SPECT acquired with ^99m^Tc-HMPAO (n = 141) or -ECD (n = 36) were included retrospectively. The vast majority of the patients was suspected to have temporal lobe epilepsy. Visual interpretation of the SPECT data was performed independently by 3 readers in 3 settings: “interictal only” (interictal SPECT and statistical hypoperfusion map), “ictal only” (ictal SPECT and hyperperfusion map), and “full” setting (side-by-side interpretation of ictal and interictal SPECT including statistical maps and SISCOM analysis). The readers lateralized the SOZ (right, left, none) and characterized their confidence using a 5-score. A case was considered "lateralizing with high confidence” if all readers lateralized to the same hemisphere with at least 4 of 5 confidence points. Lateralization of the SOZ in the “full” setting was used as reference standard.

**Results:**

The proportion of “lateralizing with high confidence” cases was 4.5/31.6/38.4% in the “interictal only”/“ictal only”/“full” setting. One (12.5%) of the 8 cases that were “lateralizing with high confidence” in the “interictal only” setting lateralized to the wrong hemisphere. Among the 56 cases that were “lateralizing with high confidence” in the “ictal only” setting, 54 (96.4%) were also lateralizing in the “full” setting, all to the same hemisphere.

**Conclusions:**

Starting brain perfusion SPECT in the presurgical evaluation of epilepsy with an interictal scan to skip the ictal scan in case of a high-confidence interictal SOZ candidate is not a useful approach. In contrast, starting with an ictal scan to skip the interictal scan in case of a high-confidence ictal SOZ candidate can be recommended.

**Supplementary Information:**

The online version contains supplementary material available at 10.1186/s13550-024-01149-8.

## Introduction

Interictal and/or ictal brain perfusion single-photon emission computed tomography (SPECT) with ^99m^Tc-labeled ethyl cysteinate dimer (^99m^Tc-ECD) [1–3] or hexamethyl-propyleneamine oxime (^99m^Tc-HMPAO) [4, 5] are widely used to identify the “onset zone” of epileptic seizures (seizure onset zone, SOZ). The SOZ is a useful indicator of the epileptogenic zone in patients with drug-resistant epilepsy to support their presurgical evaluation [6].

Numerous studies have demonstrated an ictal scan to offer higher sensitivity for the identification of the SOZ than an interictal scan (meta-analysis in [7]), especially when it is combined with the interictal scan by subtraction of ictal and interictal SPECT co-registered to MRI (SISCOM, meta-analysis in [8]). However, ictal SPECT is relatively demanding. It requires scalp video-EEG monitoring and appropriately trained staff within a structured multidisciplinary program [9]. Furthermore, ictal injection is associated with a waiting time of unpredictable duration until the next seizure. During this time, the staff must stay close to the patient to enable tracer injection as soon as possible after the seizure onset [10, 11]. Interictal SPECT is less demanding, because interictal tracer injection can be done any time sufficiently long after the last seizure [11, 12]. Thus, it might seem natural to start presurgical brain perfusion SPECT with an interictal scan. If the interictal scan provides a SOZ candidate with high confidence, the ictal scan might be omitted. This procedure requires the rate of false localization by “high-confidence” interictal SOZ candidates to be low.

Despite numerous studies on the sensitivity of ictal and/or interictal SPECT to identify the epileptogenic zone, there is a relative lack of data on the rate of false positive findings. In particular, to the best of our knowledge, the rate of false localization by interictal SOZ candidates that have been identified with high reader confidence has not yet been estimated in a large patient sample representative of clinical routine. The primary aim of the current study therefore was (1) to estimate the rate of “high-confidence” SOZ candidates in interictal brain perfusion SPECT and (2) to estimate the rate of false lateralization (to the wrong hemisphere) among these. For the latter, side-by-side interpretation of ictal and interictal SPECT with SISCOM support was used as the primary reference standard.

Conversely, the interictal scan might be skipped after ictal SPECT has identified the SOZ with high confidence, thus saving costs and reducing radiation exposure to the patient. The latter is particularly relevant due to the high proportion of children and young (≤ 60y) individuals among epilepsy patients referred to brain perfusion SPECT for presurgical evaluation. Thus, the secondary aim of this study was (1) to estimate the rate of “high-confidence” SOZ candidates in ictal brain perfusion SPECT and (2) to estimate the rate of false lateralization among them.

## Material and methods

### Patients

For this retrospective study, the database of the Department of Nuclear Medicine of the University Medical Center Hamburg-Eppendorf was searched for patients who had undergone both ictal and interictal brain perfusion SPECT for presurgical evaluation of epilepsy. The following inclusion criteria were applied: (I1) the same tracer, either ^99m^Tc-HMPAO or ^99m^Tc-ECD, was used for ictal and interictal SPECT, (I2) the same double-head camera was used for ictal and interictal SPECT, (I3) both SPECT images and structural MRI were digitally available for consistent retrospective image processing, (I4) scalp video-EEG recording during the ictal injection was available for retrospective analysis and clearly identified a seizure, (I5) the latency of ictal tracer injection after electrical seizure onset was ≤ 60 s, (I6) at the interictal tracer injection, the patient was seizure-free since at least 24 h, (I7) the time interval between both SPECT acquisitions was ≥ 48 h (in order to avoid contamination from residual radioactivity), and (I8) the time interval between ictal and interictal SPECT was ≤ 7 days (at our site, both scans are usually performed during the same inpatient stay lasting a maximum of 7 days, Monday–Monday). Severe image artifacts or technical problems hampering data interpretation (e.g., head motion during the ictal SPECT acquisition) led to exclusion. There were no eligibility criteria with respect to the suspected localization of the SOZ, MRI findings or prior therapy. This was to guarantee that the included patient sample was representative of brain perfusion SPECT for presurgical evaluation of epilepsy patients in clinical routine at our site (tertiary referral epilepsy specialist center).

The eligibility criteria led to the inclusion of 177 patients. Demographic and clinical data are summarized in Table 1. The majority of the patients had previously been included in a study on the impact of the tracer, ^99m^Tc-HMPAO versus ^99m^Tc-ECD [13], and/or in a study on the impact of the post-injection seizure duration on ictal brain perfusion SPECT [14]. A small subset of the patients had previously been included in a study on covariance pattern analysis of ictal brain perfusion SPECT for predicting the outcome of epilepsy surgery [15].

**Table 1:** Demographical and clinical data of the 177 included patients

	Median [interquartile range] (number of subjects)
Age at SPECT, y	38 [27–48]
Sex, % females	48.0
Age at first seizure, y	15 [8–26] (n = 62)
Duration of disease at SPECT, y	21 [10–31] (n = 63)
Mean seizure frequency in the last 12 months before SPECT, seizures/month	8 [4–22] (n = 50)
Time interval between ictal and interictal SPECT, d	2 [2–4]
Ictal SPECT first, %	98.8
Tracer for ictal and interictal SPECT, % ^99m^Tc-HMPAO	79.7
Injected tracer dose at SPECT, MBq, ictal/interictal	605 [510–707] (n = 118)/501 [436–560] (n = 108)
Delay between tracer injection and start of SPECT acquisition, min, ictal/interictal	141 [99–191] (n = 91)/131 [96–168] (n = 64)
Injection latency after electrical seizure onset at ictal SPECT, s	30 [23–38]
Post-injection electrical seizure duration at ictal SPECT, s	51 [30–70] (n = 161)
Alteration in structural MRI taken into consideration as epileptogenic zone, %	• Hippocampal sclerosis/atrophy (29.6%) • Border zone of prior surgery (12.0%) • Focal cortical dysplasia (11.1%) • Tumor (5.6%) • Post encephalitic lesion (1.9%) • Vessel malformation (1.9%) • Unclear lesion (13.9%) • None (24.1%) (n = 108)

Information on the seizure outcome at 12 months after epilepsy surgery was available in 44 patients. The seizure outcome was favorable (Engel outcome scale I or II [16, 17]) in 33 of these patients (75.0%). Among the 33 patients with favorable 12 months seizure outcome, surgery had been on the temporal lobe in 31 patients (93.9%, Engel I-A/B/C/D: n = 19/6/1/0, Engel II-A/B/C/D: n = 2/1/0/2). Surgery was on the frontal lobe in the remaining 2 patients (6.1%).

### SPECT imaging

Ictal and interictal injections of ^99m^Tc-ECD (n = 36) or (stabilized) ^99m^Tc-HMPAO (n = 141) were performed in an inpatient setting under scalp video-EEG monitoring in the Department of Neurology and Epileptology of the Protestant Hospital Alsterdorf. The median time interval between ictal and interictal SPECT was 2 days (interquartile range 2–4 days). Ictal SPECT was performed first in the vast majority of the patients (98.8%).

For SPECT imaging, patients were transported from the Protestant Hospital Alsterdorf to the University Medical Center Hamburg–Eppendorf (about 20 min drive). SPECT projection data were acquired for 40 min net acquisition duration with a double-head camera (either Siemens Symbia T2 or Siemens E.CAM) equipped with fan-beam or low-energy high-resolution collimators and angular steps of either 2.8° or 3.0°.

The projection data were reconstructed into transaxial SPECT images with a voxel size of 3.9 × 3.9 × 3.9 mm^3^ using filtered backprojection with a Butterworth filter of order 5 and cutoff 0.6 cycles/pixel (= 1.5 cycles/cm). The reconstructed images were postfiltered with an isotropic Gaussian kernel with 8 mm full-width-at-half-maximum. Uniform post-reconstruction attenuation correction was performed according to Chang (μ = 0.12/cm), scatter correction was not applied.

### Image preprocessing

The ictal and the interictal SPECT image of a given patient were co-registered, first to each other and then to the patient’s MRI. The Coregister tool of the Statistical Parametric Mapping software package (version SPM12) was used for this purpose. Then, both SPECT images were spatially normalized (affine) to the anatomical space of the Montreal Neurological Institute (MNI) using the Normalize tool of SPM12. The interictal SPECT was used as the object image for spatial normalization. Depending on the tracer in this patient, a custom ^99m^Tc-HMPAO template or a custom ^99m^Tc-ECD template in MNI space was used as the target for spatial normalization [13].

In preparation of voxel-based statistical testing for ictal hyperperfusion and interictal hypoperfusion, spatially normalized SPECT images were smoothed by convolution with an isotropic 3-dimensional Gaussian kernel with 15 mm full-width-at-half-maximum and then scaled to the individual mean tracer uptake in a standard cerebrum parenchyma mask predefined in MNI space. The resulting images were transformed to z-score maps relative to the voxel-wise mean and the voxel-wise standard deviation of spatially normalized, smoothed, and scaled tracer uptake in a custom age-matched ^99m^Tc-HMPAO normal database or a custom age-matched ^99m^Tc-ECD normal database, depending on the tracer in the individual patient (Supplementary Fig. 1) [11, 15]. The SPECT images in the normal databases had been acquired with the same cameras using the same acquisition and reconstruction protocol as the SPECT images included in the current analyses. The cutoff z ≥ 3.0 on the z-score was used to identify significant ictal hyperperfusion and significant interictal hypoperfusion [18]. This conservative cutoff is used in clinical routine at our site to limit the rate of false-positive clusters.

SISCOM analysis was performed using a custom approach described previously [19]. In brief, the relative difference between ictal and interictal SPECT was computed as

1 $${\text{relDiff}} = \left( {{\text{sf }}*{\text{sictal}}{-}{\text{sinterictal}}} \right) \, ./{\text{sinterictal}}$$

where “sictal” (“sinterictal”) is the 15 mm-smoothed ictal (interictal) SPECT image in MNI space, “sf” is a scale factor, and “./” denotes voxelwise division of the 3-dimensiuonal image matrices. The computation of relDiff was restricted to the standard cerebrum parenchyma mask in MNI space. The scale factor sf was determined by minimizing the sum of the squared relDiff values across all voxels in the parenchyma mask:

2 $$\sum\nolimits_{{{\text{k }}\;{\text{in }}\;{\text{mask}}}} {{\text{relDiff}}_{{\text{k}}}^{2} } \to {\text{ minimum}}.$$

The resulting relDiff image was transformed to a z-score map by scaling the relDiff map to the standard deviation (SD) of relDiff across all voxels in the parenchyma mask:

3 $${\text{z-relDiff}} = {\text{relDiff}}/{\text{SD}}.$$

For the next iteration, the scaling mask was restricted to voxels in the parenchyma mask with

4 $${\text{abs}}\left( {{\text{z-relDiff}}} \right) < {2},$$

in order to reduce the impact of ictal hyperperfusion and interictal hypoperfusion on scaling and z-score transformation. Steps (2)–(4) were repeated for up to 30 iterations. The iteration was stopped earlier if the volume of the scaling mask dropped below 300 ml or when the change in volume of the scaling mask dropped below 10 ml. The final z-relDiff map was transformed to the individual patient space using the inverse of the affine transformation from patient to MNI space. Trilinear interpolation was used to write the transformed z-redDiff map to the matrix grid in patient space. The resulting map was overlaid to the patient’s MRI. A cutoff z ≥ 2.0 on z-relDiff was used to identify significant effects in the SISCOM image [11, 20].

### Visual SPECT interpretation

**Fig. 1:**
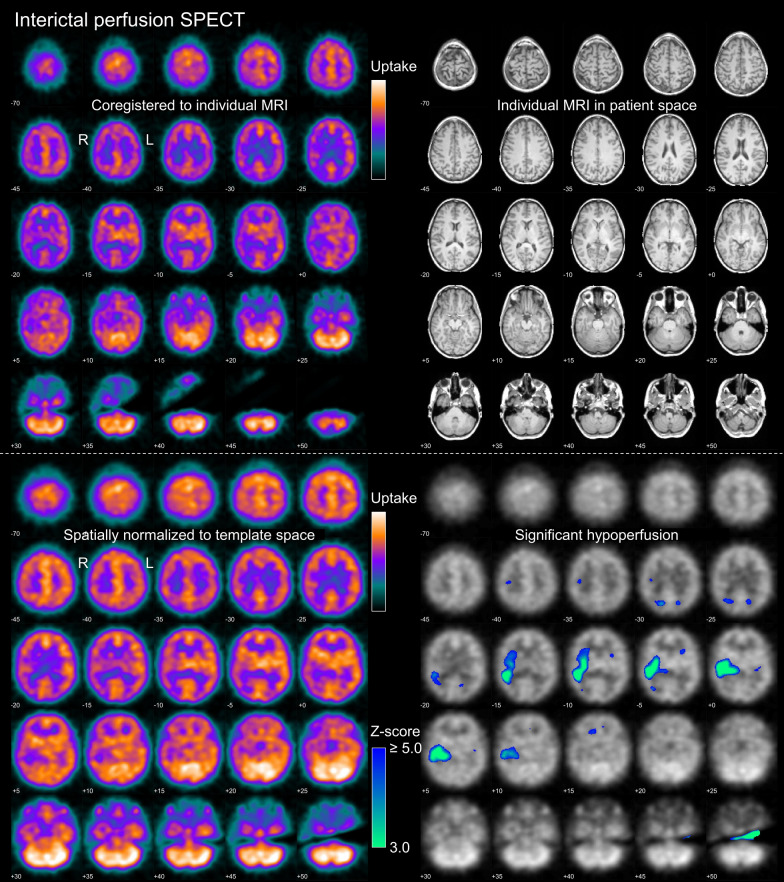
Standardized display for visual reading in the “interictal only” setting. The upper and lower parts were provided as page 1 and page 2 of a 2-page pdf-document. On the first page, the left side shows the patient’s interictal perfusion SPECT coregistered to the individual T1-weighted MRI shown on the right. On the second page, the left side shows the patient’s interictal perfusion SPECT after spatial normalization to MNI space, the right side shows the statistical hypoperfusion map thresholded at z ≥ 3.0 and overlaid to the patient’s interictal SPECT. The upper threshold of the color bar for the display of the SPECT images was set to the maximum of the 3-dimensional SPECT image volume, separately for each SPECT scan. The lower threshold of the color bar was set to zero in all cases. The example images were acquired with ^99m^Tc-HMPAO in a 28y old woman with normal MRI

**Fig. 2:**
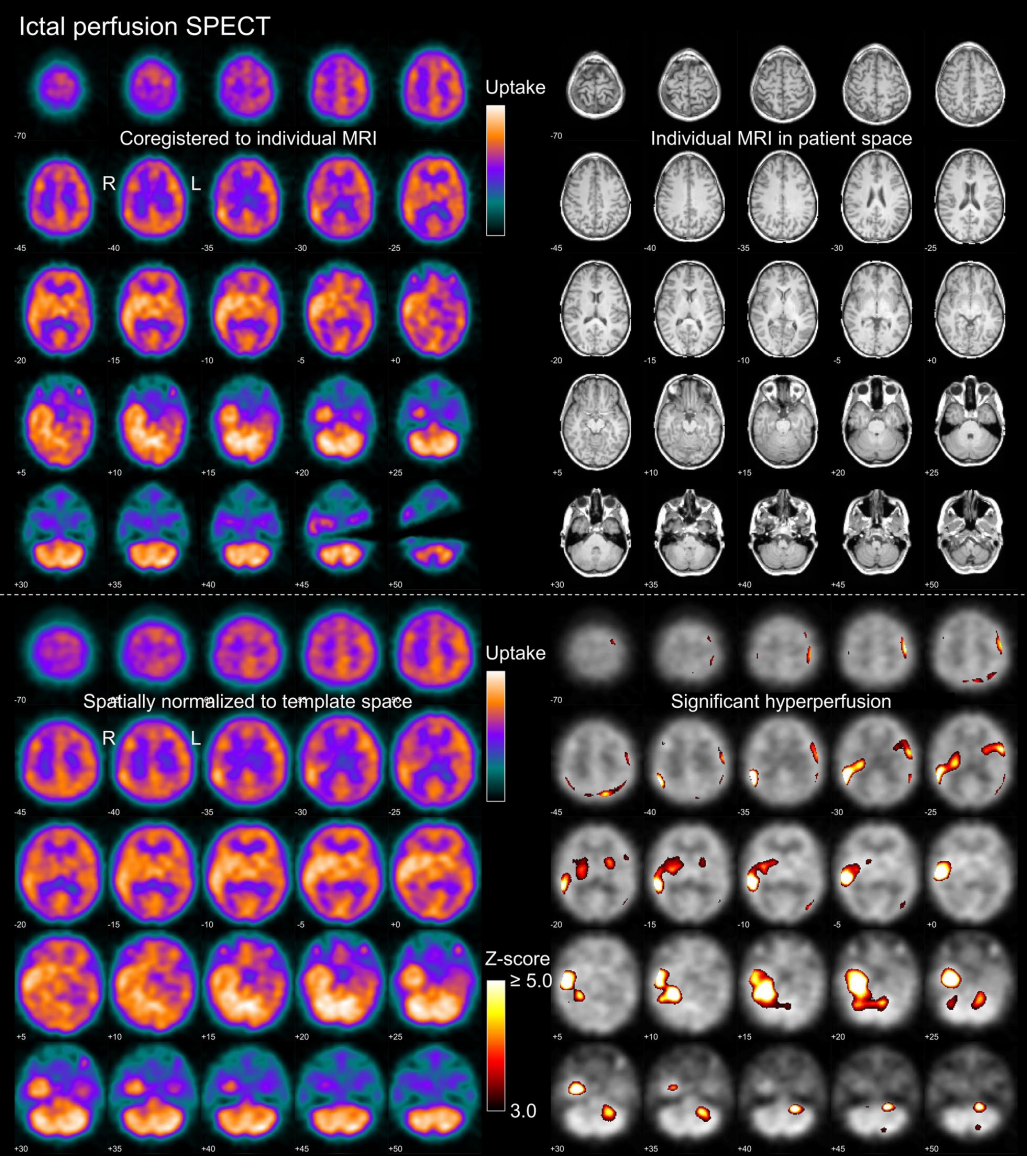
Standardized display for visual reading in the “ictal only” setting. The upper and lower parts were provided as page 1 and page 2 of a 2-page pdf-document. On the first page, the left side shows the patient’s ictal perfusion SPECT coregistered to the individual T1-weighted MRI shown on the right. On the second page, the left side shows the patient’s ictal perfusion SPECT after spatial normalization to MNI space, the right side shows the statistical hyperperfusion map thresholded at z ≥ 3.0 and overlaid to the patient’s ictal SPECT. The example images are from the same patient as in Fig. 1. Ictal SPECT was performed with 700 MBq ^99m^Tc-HMPAO injected 36 s after electrical seizure onset. The seizure continued for 70 s after the tracer injection

**Fig. 3:**
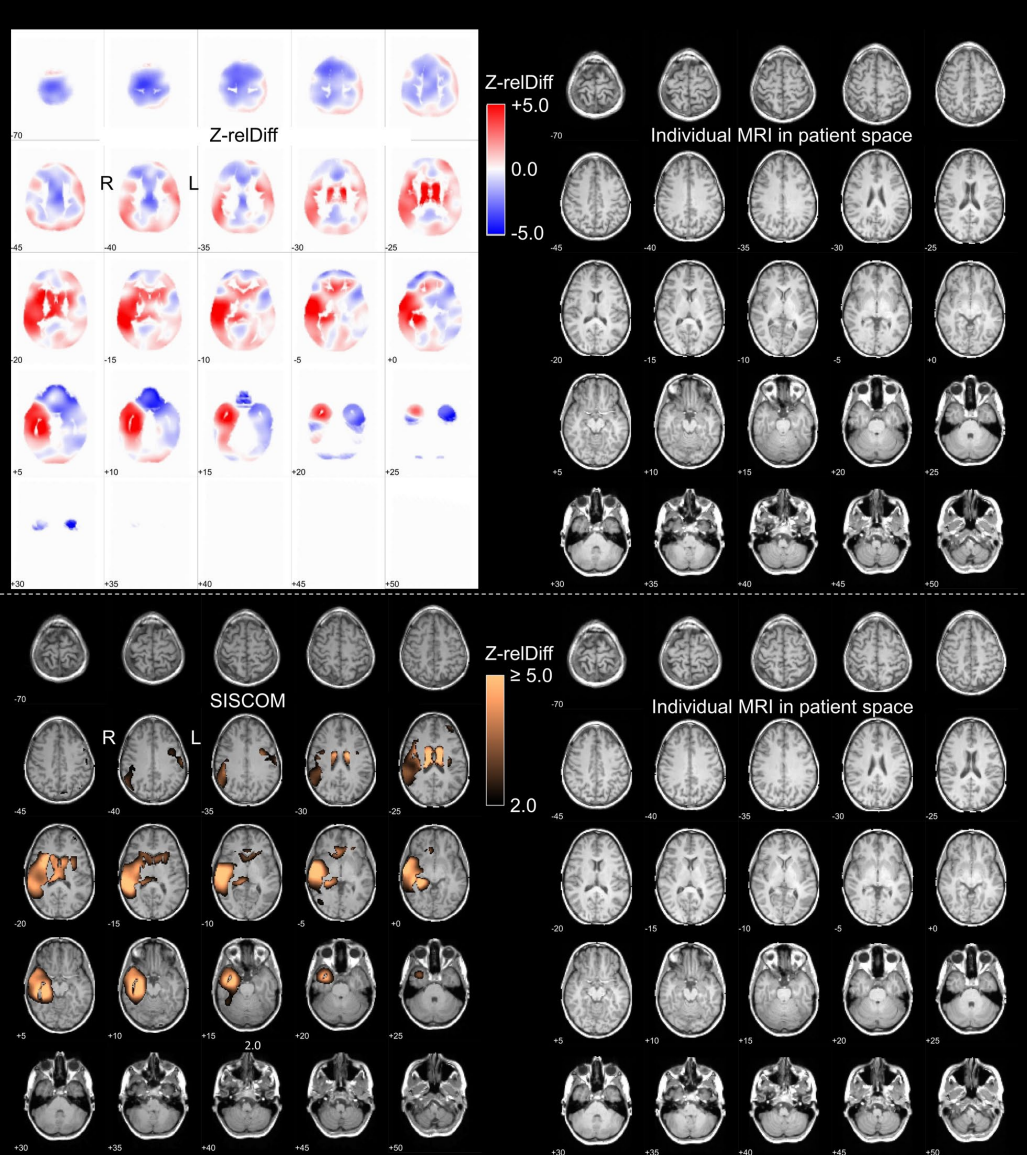
Standardized display for visual reading of SISCOM results in the “full” setting. The upper and lower parts were provided as page 7 and page 8 of an 8-page pdf-document. On page 7, the left side shows the full (non-thresholded) z-relDiff map coregistered to the individual T1-weighted MRI shown on the right. The z-relDiff map is displayed with a split color table to support the discrimination between positive (ictal > interictal) and negative (interictal > ictal) z-scores. On page 8, the left side shows the SISCOM map thresholded at z ≥ 2.0, the right side again shows the individual T1-weighted MRI. The example images are from the same patient as in Figs. 1, 2

The SPECT data were interpreted by three independent readers (KS, AK, RB) in three different settings using a standardized display for each setting: “interictal only” (Fig. 1), “ictal only” (Fig. 2) and “full” setting. The standard display for the “full” setting consisted of an 8-page pdf document starting with the two pages from the “ictal only” display (Fig. 2) followed by the two pages from the “interictal only” display (Fig. 1). The next two pages combined ictal and interictal uptake images in MNI space on one page, and the statistical maps of ictal hyper- and interictal hypoperfusion on another page (Supplementary Fig. 2). These pages aimed to simplify the side-by-side interpretation of ictal and interictal findings (no scrolling through different pages required). The last two pages displayed the full (non-thresholded) z-relDiff map and the conventional SISCOM map, that is, the z-relDiff thresholded at z ≥ 2.0 overlaid to the individual's MRI (Fig. 3).

Thus, three different pdf documents were created for each patient, resulting in a total of 531 different documents (= 177 patients * 3 settings). These documents were presented to the readers in a fully randomized order (with respect to both patient and setting) during a single reading session spread over several days. The readers were blinded to all clinical information except sex, age, and tracer, in order to avoid bias from clinical information. In particular, the readers were blinded to the suspected localization of the SOZ following standard diagnostic work-up prior to SPECT.

The readers were asked to first lateralize the SOZ: right hemisphere, left hemisphere, or no evidence of the SOZ. If a reader lateralized the SOZ to the right or to the left hemisphere, she/he first rated her/his confidence regarding the lateralization according to a 5 point scale (1 = very low confidence,…, 5 = very high confidence) and then localized the SOZ within a brain lobe (temporal, frontal, parietal, occipital) in the selected hemisphere. The category “no evidence of SOZ” was restricted to cases with normal tracer uptake. In cases with ≥ 2 SOZ candidates considered equally likely to represent the epileptogenic zone, the readers were asked to select one SOZ candidate and, if applicable, express their uncertainty by a low confidence score.

One of the readers had about 20 years of previous experience in reading brain perfusion SPECT in the presurgical evaluation of epilepsy patients. The other two readers did not have experience in reading brain perfusion SPECT before this study. In order to account for this, the readers were asked to adhere to the detailed recommendations for visual interpretation given in section “Criteria for visual identification of the seizure onset zone” in the supplementary material. In addition, the readers were provided with a figure presenting the voxel-wise mean and the voxel-wise coefficient of variance in the custom ^99m^Tc-ECD and ^99m^Tc-HMPAO normal databases as reference for the normal distribution pattern of these tracers in the brain (Supplementary Fig. 1). Before reading the 531 pdf documents for the current study, each reader underwent a training session with 12 randomly selected cases. The results of this training session were discussed among the readers to reduce between-readers variability.

### Statistical analysis

Proportions are given as percentages, continuous variables are characterized by their median and interquartile range.

Between-readers agreement of the lateralization of the SOZ (right hemisphere, left hemisphere, no SOZ candidate) was assessed by Fleiss’ kappa (κ), separately for each setting. The statistical significance of pairwise κ differences between settings was assessed by checking the 83.4% confidence intervals (CI) for the corresponding κ estimates for overlap (non-overlapping 83.4% CIs indicate statistical significance with a 5% type 1 error probability) [21]. The 83.4% CI was computed as κ ± 1.385 * standard error [22].

To test for reader-independent between-setting effects on the lateralization of the SOZ, the lateralization scores and the lateralization confidence scores of the three readers were combined into an overall “lateralizing” 3-score: “non-lateralizing”, “lateralizing with low confidence”, and “lateralizing with high confidence”. A case was considered "lateralizing with high confidence” if the three readers agreed on the lateralization of the SOZ (either right or left hemisphere) and each reader scored her/his confidence regarding the lateralization with ≥ 4 of 5 points on the confidence scale. A case was considered "lateralizing with low confidence” if the three readers agreed on the lateralization of the SOZ (either right or left hemisphere) and at least one reader rated her/his lateralization confidence with less than 4 points. All other cases were considered “non-lateralizing” (including cases scored as “no SOZ candidate” by at least one reader and cases with disagreement among the readers regarding the hemisphere). The impact of the setting on the proportion of “non-lateralizing”, “lateralizing with low confidence”, and “lateralizing with high confidence” cases was tested by repeated measures analysis of variance (ANOVA) with the lateralizing 3-score as dependent variable and setting (“interictal only”, “ictal only”, “full”) as within-subjects factor. Post-hoc testing of pairwise differences between two settings was performed with repeated measures ANOVA restricted to those two settings. Patient-level between-settings differences of the lateralizing 3-score were assessed by 3 × 3 cross tables.

A case was considered “localizing” if it was lateralizing (with low or high confidence) and the three readers also agreed on the same lobe within the same hemisphere. The primary aim of the localization was to characterize the patient sample with respect to temporal versus extratemporal SOZ. The localization analysis therefore was restricted to the “full” setting.

Finally, SOZ lateralization was correlated with the operated hemisphere in the 31 patients with favorable seizure outcome 12 months after temporal lobe epilepsy surgergy, separately for each setting.

The statistical analysis was performed with SPSS (version 29). An effect was considered statistically significant if two-sided *p* < 0.05.

## Results

### Between-readers agreement of the lateralization

Fleiss’ between-readers κ of the SOZ lateralization (right hemisphere, left hemisphere, no SOZ candidate) was significantly lower (non-overlapping 83.4% CI) in the “interictal only” setting (0.517, 83.4% CI 0.473–0.561) compared with both, the “ictal only” setting (0.715, 0.661–0.769) and the “full” setting (0.768, 0.718–0.818). The difference between “ictal only” and “full” setting was not significant (overlapping 83.4% CI).

### Between-settings differences of the lateralizing 3-score

**Fig. 4:**
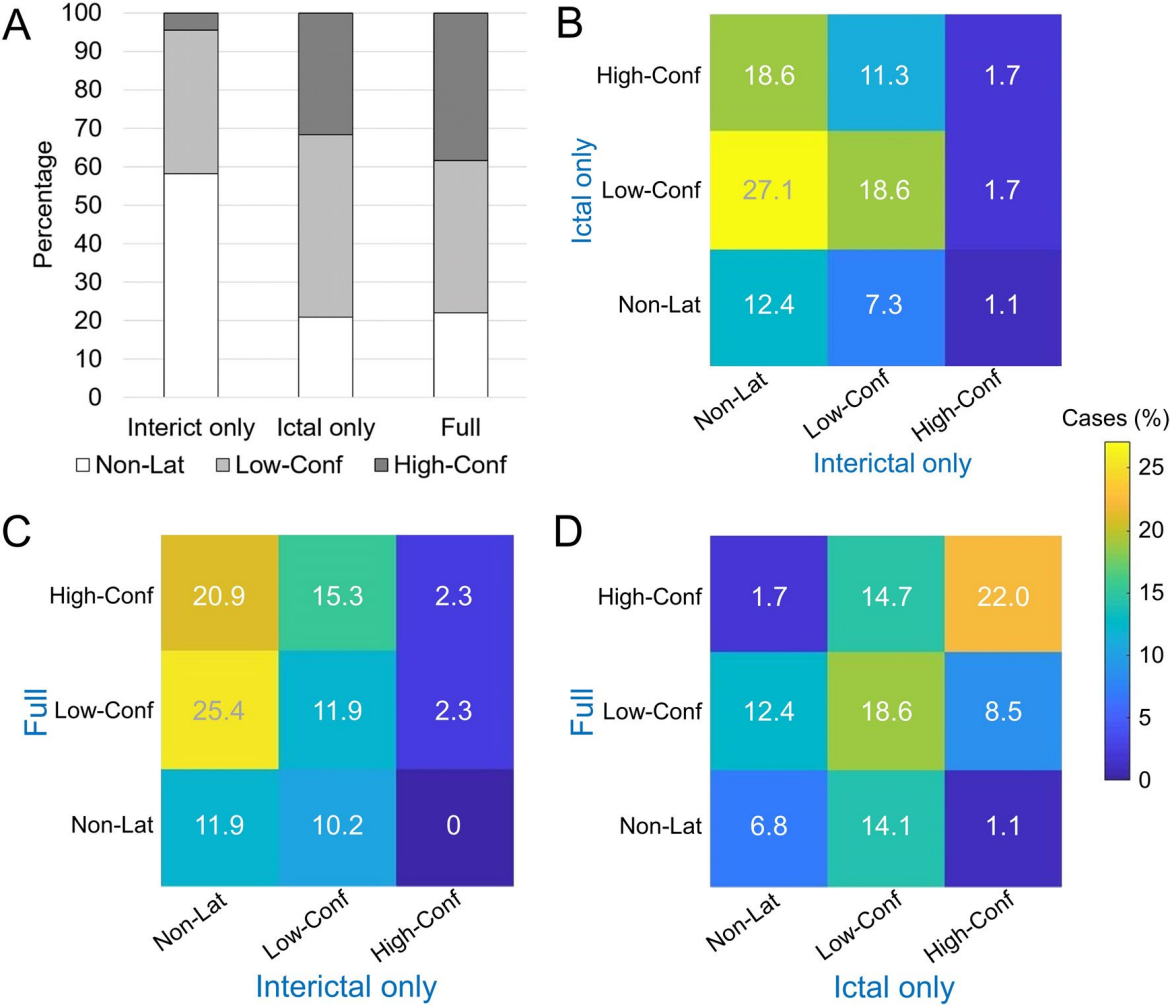
Between-settings differences of the lateralizing 3-score. **A** Proportion of “non-lateralizing” (Non-Lat), “lateralizing with low confidence” (Low-Conf) and “lateralizing with high confidence” (High-Conf) cases in each of the three settings (“interictal only”, “ictal only”, “full”). **B**–**D** Cross-tables of the lateralizing 3-score for each pair of settings. The numbers represent the percentages of cases relative to the whole patient sample (n = 177)

The proportion of “lateralizing with high confidence” cases was 4.5/31.6/38.4% in the “interictal only”/“ictal only”/“full” setting, the proportion of “lateralizing with low confidence” cases was 37.3/47.5/39.5%, and the proportion of “non-lateralizing” cases was 58.2/20.9/22.0% (Fig. 4A). The difference of the lateralizing 3-score in the “interictal only” setting was statistically significant compared with both, the “ictal only” and the “full” setting (both *p* < 0.001). The difference between the “ictal only” and the “full” setting was not significant (*p* = 0.337).

The overall lateralization rate (independent of the confidence) was 41.8/79.1/77.9% in the “interictal only”/“ictal only”/“full” setting.

Cross-tables of the lateralizing 3-score for each pair of settings are shown in Fig. 4B-D.

### Concordant and discordant lateralization relative to the “full” setting

**Fig. 5:**
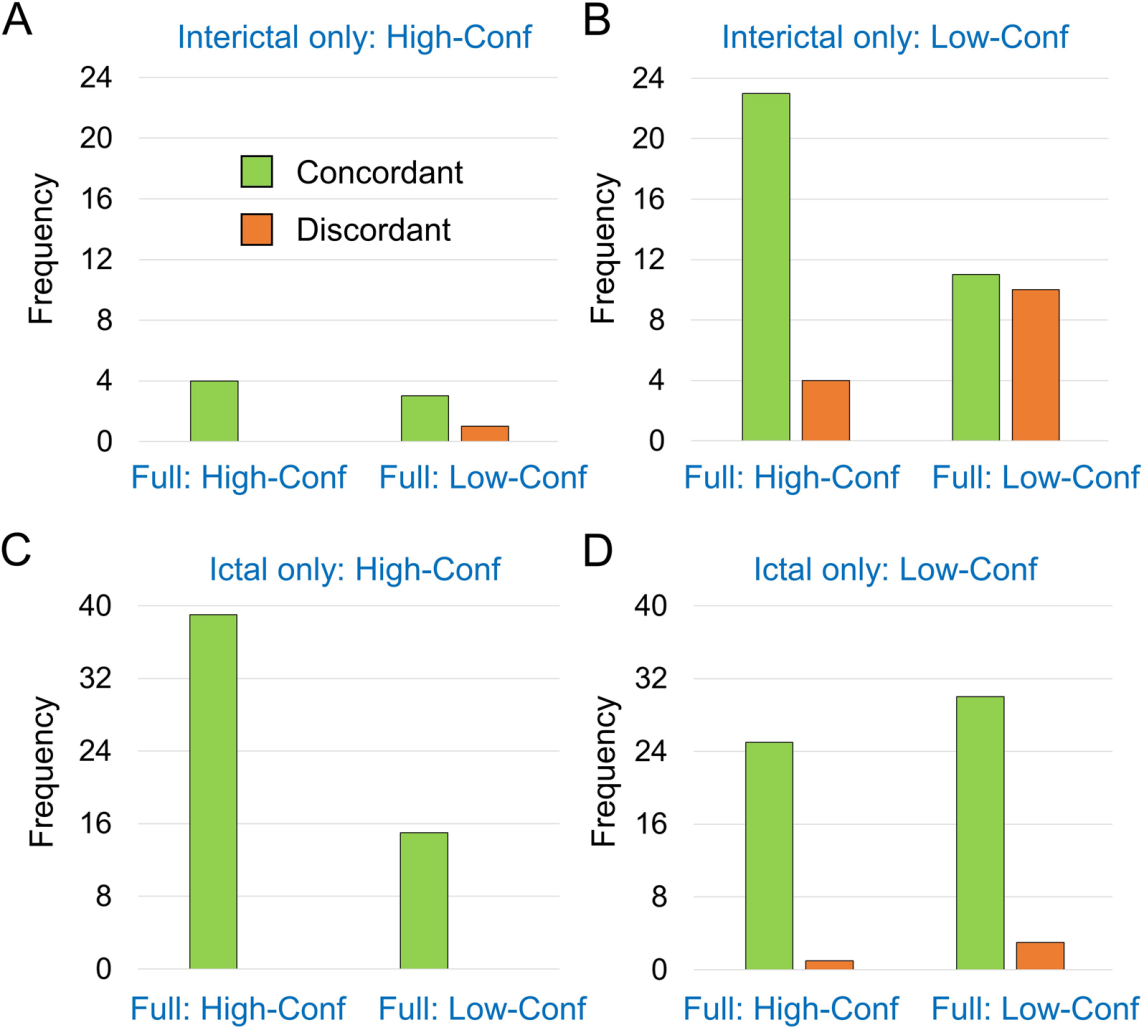
Concordant and discordant lateralization relative to the “full” setting. Frequency (number of cases) of concordant lateralization (same hemisphere) and discordant lateralization (different hemispheres) compared with the “full” setting, separately for the “interictal only” setting (**A**, **B**) and the “ictal only” setting (**C**, **D**), and separately for “lateralization with high confidence” (**A**,** C**) and “lateralization with low confidence” (**B**,** D**). In each of the subplots (**A**–**D**), the cases are sorted according to the confidence regarding the lateralization in the “full” setting (left: high confidence, High-Conf; right: low confidence, Low-Conf). Cases that were not lateralizing in the “full” setting are excluded (Fig. 4C, D). The scaling of the vertical axis differs between the “interictal only” setting (**A, B**: 0–24) and the “ictal only” setting (**C, D**: 0–40) to account for the lower number of lateralizing cases in the “interictal only” setting

The frequency of concordant lateralization (same hemisphere) and discordant lateralization (different hemispheres) compared with the “full” setting is shown in Fig. 5, separately for the “interical only” setting (parts A, B) and the “ictal only” setting (C, D), and separately for “lateralizing with high confidence” (A, C) and “lateralizing with low confidence” (B, D).

#### Interictal only

**Fig. 6:**
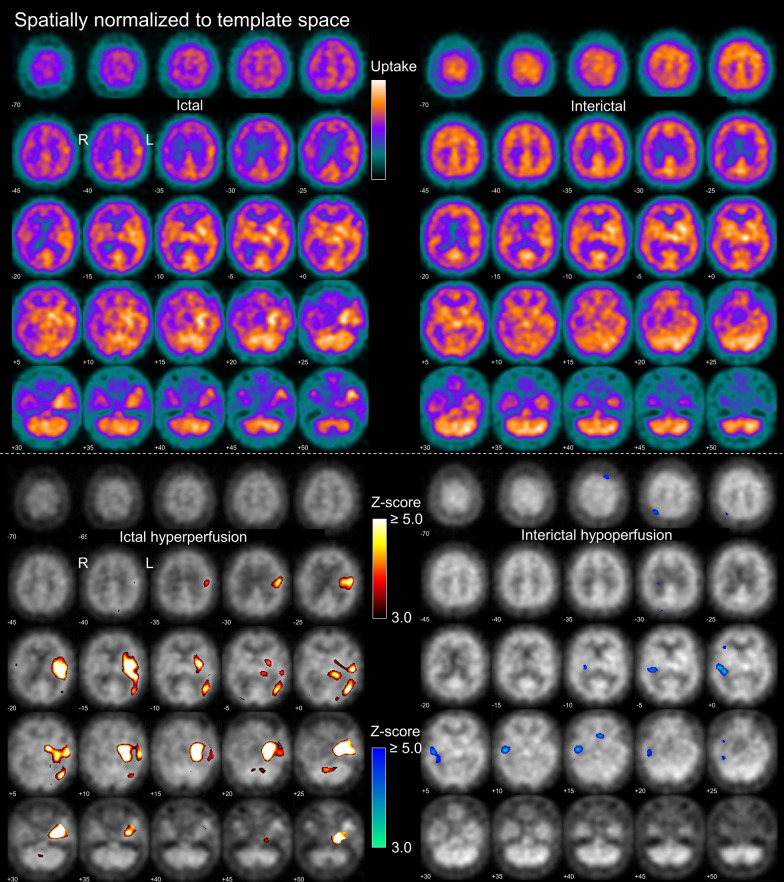
False lateralization by interictal SPECT. Ictal SPECT and interictal SPECT with.^99m^T-HMPAO in a 29 y old man with therapy refractory epilepsy and unclear lesion in the left temporal lobe (ictal SPECT: injection of 569 MBq 33 s after electrical seizure onset, post-injection seizure duration 40 s, start of SPECT acquisition 91 min after injection; interictal SPECT: injection of 534 MBq, start of SPECT acquisition 93 min after injection). In the ictal SPECT, the SOZ was lateralized to the *left* hemisphere with highest possible confidence (confidence score = 5 by all readers). In the interictal SPECT, the SOZ was lateralized to the *right* hemisphere with high confidence (confidence score = 4/5 by 1/2 readers). In the “full” setting, the SOZ was lateralized to the *left* hemisphere with low confidence (confidence score = 3/5 by 1/2 readers)

All eight cases that were “lateralizing with high confidence” in the “interictal only” setting were also lateralizing in the “full” setting, four (50%) of them “with high confidence” and four (50%) with “low confidence” (Fig. 4C). Seven (87.5%) of these eight cases were lateralizing to the same hemisphere in both settings (Fig. 5A). One case (12.5%) was “lateralizing with high confidence” to the *right* hemisphere in the “interictal only” setting and “lateralizing with low confidence” (confidence score = 3/5 in 1/2 readers) to the *left* hemisphere in the “full” setting (Fig. 6).

Among the 66 cases that were “lateralizing with low confidence” in the “interictal only” setting, 18 (27.3%) were “non-lateralizing” in the full setting (Fig. 4C). The remaining 48 (72.7%) were also lateralizing in the “full” setting, 21 (43.8%) of them with low confidence and 27 (56.3%) with high confidence. Among these 48 cases, “interictal only” and “full” lateralization were concordant in 34 cases (70.8%), while discordance was noted in 14 cases (29.2%) (Fig. 5B).

#### Ictal only

Among the 56 cases that were “lateralizing with high confidence” in the “ictal only” setting, 54 (96.4%) were also lateralizing in the “full” setting, 39 (69.6%) “with high confidence” and 15 (26.8%) “with low confidence” (Fig. 4D). All of these 54 cases (100%) were lateralizing to the same hemisphere in the “ictal only” and in the “full” setting (Fig. 5C).

Among the 84 cases that were “lateralizing with low confidence” in the “ictal only” setting, 25 (29.8%) were “non-lateralizing” in the full setting (Fig. 4D). The remaining 59 (70.2%) were also lateralizing in the “full” setting, 33 (55.9%) of them with low confidence and 26 (44.1%) with high confidence. Among these 59 cases, “ictal only” and “full” lateralization were concordant in 55 cases (93.2%), while discordance was noted in four cases (6.8%) (Fig. 5D).

### Localization of the SOZ in the “full” setting

111 of the 177 cases (62.7%) were “localizing”, 107 (96.4%) of them to the temporal lobe. The remaining four “localizing” cases were localizing to the parietal (n = 2, 1.8%), frontal (n = 1, 0.9%) or occipital (n = 1, 0.9%) lobe.

### Correct and false lateralization relative to favorable surgical outcome

**Fig. 7:**
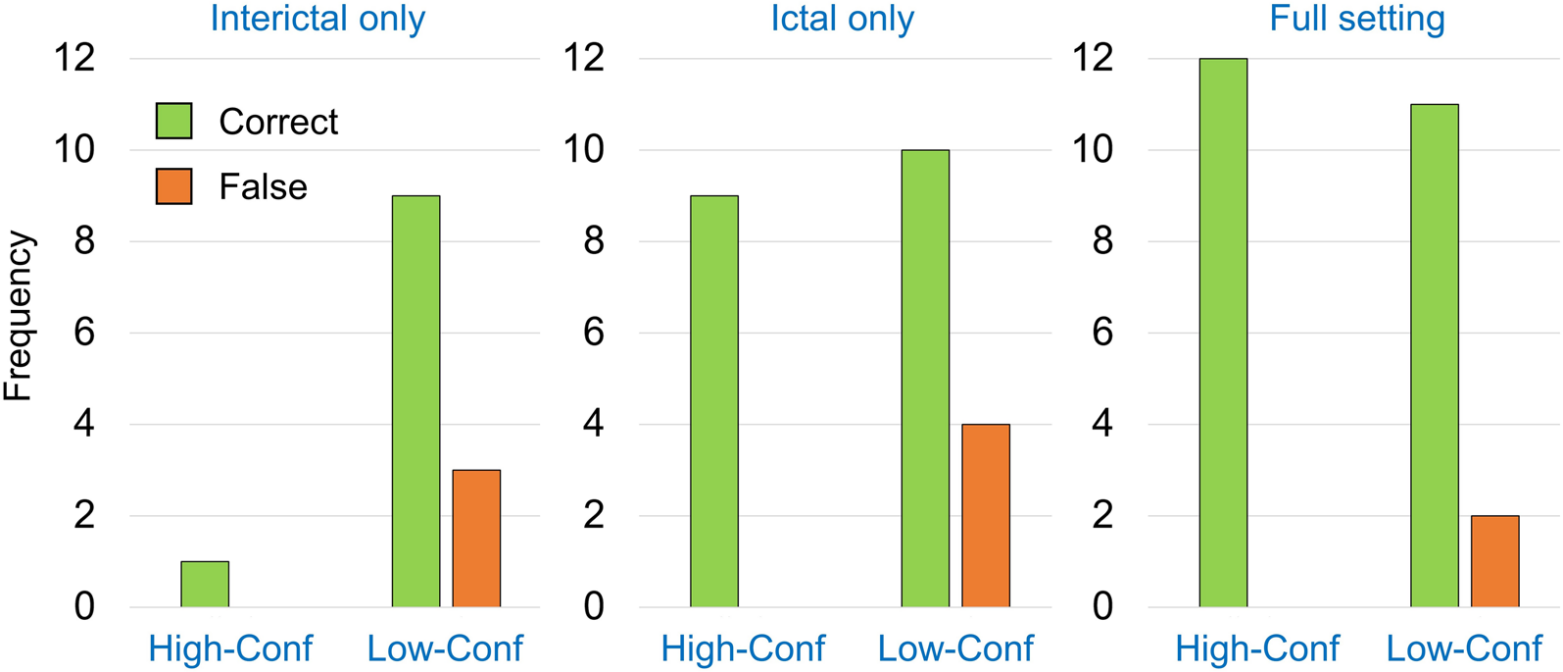
Lateralization accuracy relative to favorable surgical outcome. Frequency (number of cases) of correct lateralization (to the operated hemisphere, green) and false lateralization (contralateral to the operated hemisphere, orange) in 31 patients with favorable seizure outcome (Engel I or II) 12 months after temporal lobe surgery. The frequencies are shown separately for each of the 3 considered settings (“interictal only”, “ictal only”, “full” setting) and separately for “lateralization with high confidence” and “lateralization with low confidence” (left: high confidence, High-Conf; right: low confidence, Low-Conf). Non-lateralizing cases were excluded, separately for each setting

The lateralization accuracy in the 31 patients with favorable 12 months seizure outcome of temporal lobe surgery is summarized in Fig. 7. All cases with high-confidence lateralization were correct in each of the 3 settings (“interical only”: n = 1, “ictal only”: n = 9, “full” setting: n = 12).

## Discussion

The aim of the current study was to estimate the added value of ictal SPECT after high-confidence SOZ lateralization by interictal SPECT and vice versa. Side-by-side interpretation of ictal and interictal SPECT supported by SISCOM analysis was used as reference standard. The rationale for this was that side-by-side interpretation of ictal and interictal SPECT supported by SISCOM (“full” setting) is generally considered the gold standard for lateralization and localization of the SOZ by brain perfusion SPECT. Under this assumption, the added value of ictal (interictal) SPECT after high-confidence SOZ lateralization by interictal (ictal) SPECT is the smaller the higher the agreement of high-confidence “interictal only” (“ictal only”) lateralization with the “full” setting lateralization.

The study was focused on lateralization (rather than localization) performance, because the vast majority of the included patients had temporal lobe epilepsy. This is in line with previous studies on the utility of brain perfusion SPECT in temporal lobe epilepsy that focused on the lateralization of the SOZ [23].

The primary finding of the study was that the proportion of interictal SPECT scans that were “lateralizing with high confidence” was notably small, only 4.5%, and that one (12.5%) of these cases was lateralizing to the wrong hemisphere (with reference to the “full” setting). This finding was corroborated by the only moderate between-readers agreement of the SOZ lateralization in the interictal SPECT scans and by the poor agreement of the lateralizing 3-score between the “interictal only” and the “full” setting. Among the 31 patients with favorable surgical outcome, high-confidence interictal lateralization was correct. However, this occurred in a single case only, which limits its significance. Taking these findings together, starting brain perfusion SPECT in the presurgical evaluation of epilepsy with an interictal scan and then skipping the ictal scan in case of a “high-confidence” interictal SOZ candidate is not a sensible approach, in line with EANM practice guidelines [11]. The current study adds to the EANM guidelines by demonstrating that interictal SPECT alone is not sufficiently reliable even if it provides a “high-confidence” SOZ candidate.

The rather large proportion on non-lateralizing interictal SPECT (58.2%) in the current study is in agreement with the meta-analytic 44% sensitivity of interictal SPECT in temporal lobe epilepsy [7]. The 12.5% estimate for the rate of false lateralization by “lateralizing with high confidence” interictal SOZ candidates is affected by a rather large error margin due to the small number of “lateralizing with high confidence” interictal SPECT. Taking this into account, the observed rate of false lateralization is in line with the meta-analytic rate of false lateralization of interictal SPECT (independent of the readers’ confidence) of 9.5% relative to EEG and 5.5% relative to good outcome of epilepsy surgery [7].

The secondary finding of the current study was that almost one third of the ictal SPECT was “lateralizing with high confidence” and that none of these ictal SPECT lateralized to the other hemisphere compared with the “full” setting. This finding was corroborated by the fact that among the patients with favorable surgical outcome, all high-confidence ictal lateralizations were correct (to the operated hemisphere, Fig. 7). Thus, starting brain perfusion SPECT in the presurgical evaluation of epilepsy with an ictal scan and then skipping the interictal scan in case of a “high confidence” ictal SOZ candidate is a clinically useful approach. It allows saving costs and radiation exposure of an interictal scan in about one third of the patients.

In contrast, “lateralization with low confidence” in ictal SPECT did not agree with the “full” setting reference standard in about one third of these cases: about 30% of these cases were non-lateralizing in the “full” setting (Fig. 4D) and about 5% were lateralizing to the other hemisphere (Fig. 5D). Furthermore, the correlation with favorable surgical outcome as reference standard revealed almost one third of the ictal low-confidence lateralizations among the patients with favorable surgical outcome to be false (contralateral to the operated hemisphere, Fig. 7). Thus, skipping the interictal SPECT after an ictal SPECT that is “lateralizing with low confidence” cannot be recommended.

The level of experience in reading brain perfusion SPECT images in the presurgical evaluation of epilepsy was very different between the 3 independent readers in the current study. Whereas one reader had about 20 years of experience, the other two readers did not have any experience in reading brain perfusion SPECT before this study. However, Fleiss’ between-readers κ of the SOZ lateralization according to 3 categories (right hemisphere, left hemisphere, no SOZ candidate) was 0.768, 95%-confidence interval 0.718–0.818, in the “full” setting. This indicates substantial to almost perfect agreement [24], suggesting that the measures to avoid excessive between-readers variability due to varying experience levels were rather effective (subsection “Visual SPECT interpretation” in Materials and Methods). This might be explained by the fact that support of visual reading of nuclear medicine brain images by voxel-based statistical maps (such as the thresholded statistical hyper- or hypoperfusion maps and the thresholded SISCOM maps) often enables beginners to achieve very similar performance than expert readers after a brief training session [25].

Repeat analyses using the visual interpretation by the experienced reader alone revealed essentially the same relationship between the 3 considered settings (“interictal only”, “ictal only”, “full”), both among each other and with the favorable surgical outcome as reference, as the analyses based on the lateralizing 3-score combining the 3 independent readers (section “Repeat analyses restricted to the experienced reader” and Supplementary Figs. 3, 4 and 5 in the supplementary material). This suggests that the findings of the current study regarding its primary aim, the added value of ictal SPECT after high-confidence lateralization of the SOZ by interictal SPECT and vice versa, are not limited by the varying experience of the readers.

The “logistic” problems of ictal brain perfusion SPECT associated with the unpredictable duration of the waiting period for a spontaneous seizure might be avoided by intentionally provoking seizures. Calcagni and co-workers proposed infusion of pentylenetetrazol to provoke a seizure pharmacologically [26]. A typical seizure was observed within 2 to 20 min after start of the infusion in about 80% of 50 patients without any adverse events. Injection of 740 MBq ^99m^Tc-ECD 5 to 7 s after seizure onset resulted in regional hyperperfusion in the area of interictal hypoperfusion in about 80% of these patients. Among 19 of these patients that underwent ablative surgery, 17 were seizure-free at 3 years follow-up. The authors concluded that with this “feasible procedure it is possible to localise the focus, to avoid the limitations due to the unpredictability of seizures, to avoid pitfalls due to late injection, to avoid intracranial EEG recording and to minimize costs” [26]. The utility of ictal SPECT during pentylenetetrazol-provoked seizures was confirmed by further studies of the same group [27, 28]. Enev and co-workers proposed electroconvulsive therapy to induce typical generalized tonic–clonic seizure resembling spontaneous generalized seizures [29]. Very recently, Barlatey and co-workers reported a feasibility study on triggering patient-typical seizures within 3–8 min by direct stimulation of epileptic networks via stereotactic EEG electrodes [30].

Limitations of the current study include the following. First, clinical follow-up of ≥ 12 months after surgery was available in about one fourth of the patients only. Thus, the analyses with favorable surgical outcome as reference standard are limited by reduced sample size. Second, the ictal SPECT was performed before the interictal SPECT in the vast majority (98.8%) of the included patients, because this is the standard procedure at our site. A retrospective search of our database for all patients with ictal and/or interictal SPECT revealed that ictal SPECT was complemented by interictal SPECT in about 80% of the cases. In the remaining 20% of cases, interictal SPECT had been cancelled, either because the ictal findings were considered sufficiently clear so that no added value was expected from an interictal SPECT (in about half of these cases), or because ictal SPECT had not revealed a SOZ candidate. The exclusion of these cases from the current analyses (due to missing interictal SPECT) might have caused a selection bias. However, the potential selection bias primarily affects the performance estimates for ictal SPECT. The performance estimates for interictal SPECT (the primary aim of the study) most likely are not affected. The search of our database identified only very few epilepsy patients (< 2%) with interictal but no ictal SPECT. This is explained by the fact that interictal only imaging is performed with brain [^18^F]fluorodeoxyglucose PET at our site. Thus, there was no selection bias regarding the interictal SPECT data included in this study. Third, about 95% of the “localizing” cases localized the SOZ to the temporal lobe and about 95% of the patients with favorable surgical outcome had been operated on the temporal lobe. This suggests that the vast majority of the included patients had temporal lobe epilepsy. The current findings might not be translated without change to extratemporal epilepsies [31]. Fourth, spatial registration of the SPECT images to the anatomical reference space in preparation of voxel-based statistical testing was performed by SPECT-based 12 parameters affine registration (translation, rotation, spatial scaling, shear), although non-linear (including warping) MRI-based spatial registration is the gold standard for spatial registration of nuclear medicine brain images (and individual MRI was digitally available for the retrospective analyses in all patients included in the current study). The rationale for using SPECT-based registration was that in clinical practice, the most recent MR images are not always digitally available to us at the time of SPECT imaging. However, we do not consider this a limitation of the study, as perfusion-SPECT images provide sufficient anatomical information for SPECT-based registration to be reliable. Affine registration was used, because high-dimensional non-linear spatial registration does not provide an added value in conventional brain perfusion SPECT (limited spatial resolution and rather high levels of statistical noise), neither with respect to visual interpretation nor with respect to voxel-based testing. The differences in the registered images between affine and non-linear transformation are so small that they are hardly detectable visually even by careful side-by-side inspection. Strong spatial smoothing of the registered images in preparation of voxel-based testing (by convolution with an isotropic 3-dimensional Gaussian kernel with 15 mm full-width-at-half-maximum in the current study) eliminates the small differences prior to voxel-based testing. Finally, the visual analysis of the SPECT images adhered to the common procedure guidelines for brain perfusion SPECT in epilepsy [10, 11, 32–35] except for the use of standardized pdf-documents rather than a computer monitor. Thus, interactive manipulations such as changing the color table or the image orientation was not possible. The rationale for the use of the pdf-documents was to standardize the reading across the readers. Furthermore, different than in practice, the readers were intentionally blinded to clinical and EEG data to avoid a potential bias.

In conclusion, interictal SPECT allowed lateralization of the SOZ with high confidence in only about 5% of the patients, and even in these cases a rate of about 10% false lateralization was observed. Thus, starting brain perfusion SPECT in the presurgical evaluation of epilepsy with an interictal scan and then skipping the ictal scan in case of a “high-confidence” interictal SOZ candidate is not a useful approach. In contrast, ictal SPECT allowed lateralization of the SOZ with high confidence in about one third of the patients without any false lateralization (relative to the “full” setting and relative to favorable surgical outcome as reference standard). Thus, starting brain perfusion SPECT with an ictal scan and then skipping the interictal scan in case of a “high-confidence” ictal SOZ candidate can be recommended.

## Supplementary Information

Additional file 1.

## Data Availability

The datasets supporting the conclusions of this article can be made available on request.

## Abbreviations

^99m^Tc-ECD: ^99M^Tc-labeled ethyl cysteinate dimer
^99m^Tc-HMPAO: ^99M^Tc-labeled hexamethyl-propyleneamine oxime
ANOVA: Analysis of Variance
CI: Confidence interval
EEG: Electroencephalography
MNI: Montreal Neurological Institute
MRI: Magnetic resonance imaging
PET: Positron emission tomography
SISCOM: Subtraction ictal SPECT coregistered to MRI
SOZ: Seizure onset zone
SPECT: Single-photon emission computed tomography
SPM12: Statistical Parametric Mapping software package, version 12
κ: Fleiss’ or Cohen’s kappa
