# Supplementary material for: Brain perfusion SPECT in the presurgical evaluation of epilepsy: is additional ictal SPECT required in case of high-confidence lateralization of the seizure onset zone by interictal SPECT and vice versa?
Source: EJNMMI Res. 2024 Sep 12;14:83. doi: 10.1186/s13550-024-01149-8 (PMC11393367; doi:10.1186/s13550-024-01149-8)
Supplement: Supplementary file 1 — Additional file 1. [file 13550_2024_1149_MOESM1_ESM.docx]

**Supplementary material**

**Brain perfusion SPECT in the presurgical evaluation of epilepsy: is additional ictal SPECT required in case of high-confidence lateralization of the seizure onset zone by interictal SPECT and vice versa?**

Kian Baradaran-Salimi^1^, Amir Karimzadeh^1^, Berthold Voges^2^, Ivayla Apostolova^1^, Thomas Sauvigny^3^, Olga Simova^2^, Michael Lanz^2^, Susanne Klutmann^3^, Stefan Stodieck^2^, Philipp T. Meyer^4^, Ralph Buchert^1^

Departments of ^1^Diagnostic and Interventional Radiology and Nuclear Medicine and ^3^Neurosurgery, University Medical Center Hamburg-Eppendorf, Hamburg, Germany

^2^Department of Neurology and Epileptology, Protestant Hospital Alsterdorf, 22337 Hamburg, Germany

^4^Department of Nuclear Medicine, Medical Center - University of Freiburg, Faculty of Medicine, University of Freiburg, Freiburg, Germany

*These authors contributed equally as first authors

1. **Criteria for visual identification of the seizure onset zone**

## **Ictal SPECT**

- the SOZ is identified by regional *hyper*perfusion in cortical grey matter according to visual inspection of the uptake image and/or the statistical parametric map of ictal *hyper*perfusion
- the other way round is not true, that is, regional hyperperfusion in cortical grey matter is not automatically to be interpreted as the SOZ. For example, hyperperfusion in the insula and in the basal ganglia is often caused by seizure propagation from a SOZ in the ipsilateral temporal lobe. Hyperperfusion in the motor cortex and the supplementary motor area also might be secondary effects of the seizure and, therefore, might not indicate the SOZ
- there often is a “mirror focus” of the SOZ, that is, less pronounced hyperperfusion in the homologous brain region in the other hemisphere. In case of temporal seizures, the mirror focus often is restricted to the lateral part of the temporal cortex and is associated with less pronounced hyperperfusion in the insula, striatum, and thalamus in the hemisphere of the mirror focus. A mirror focus can increase the confidence regarding the localization of the SOZ within a specific brain lobe. However, it often reduces the confidence regarding the lateralization
- the SOZ might be identified in the uptake image only, that is, it is not required that the SOZ is confirmed by a cluster of significant hyperperfusion in the statistical parametric map (the rather conservative threshold of 3.0 z-score points is expected to result in some false negative findings). In particular, the SOZ might be identified by considerable left-right asymmetry in the uptake image. For example, a SOZ in the left temporal lobe might be identified by higher tracer uptake in the left temporal lobe compared to the right temporal lobe, although the (relative) increase of ictal tracer uptake in the left temporal lobe does not reach the level of statistical significance and, therefore, is not confirmed by a significant cluster in the statistical parametric map
- identification of the SOZ might also be based primarily on a cluster of significant hyperperfusion in the statistical parametric map, if on visual inspection of the uptake image the regional hyperperfusion does not appear very prominent (the conservative threshold of 3.0 z-score points is expected to result in a rather low rate of false positive findings)
- a statistically significant cluster of regional hyperperfusion located in white matter should not be interpreted as SOZ. It could be caused by mismatch of spatial resolution between the patient image and the normal database used for voxel-based testing, particularly when located close to the white-to-gray matter junction
- a statistically significant cluster of regional hyperperfusion located in white matter can extend into cortical grey matter due to partial volume effects caused by the limited spatial resolution of SPECT. This does not indicate hyperperfusion in cortical grey matter and, therefore, should not be interpreted as SOZ
- regional hyperperfusion in the cerebellum should not be interpreted as SOZ
- the ictal perfusion pattern in the cerebellum does not provide reliable information to support lateralization or localization of SOZ candidates in the cerebrum [1, 2]
- strongly pronounced regional hyperperfusion is usually associated with higher confidence that this region is the SOZ than mild hyperperfusion
- clear ictal hyperperfusion in the boundary of a (surgical) defect is associated with high confidence, because the actual hyperperfusion most likely is considerably underestimated due to partial volume effects
- the confidence that regional ictal hyperperfusion indicates the SOZ might be reduced if there is a defect or lesion in the homologous region in the contralateral hemisphere: the detected regional hyperperfusion might be a mirror focus due to propagation from the border zone of the defect/lesion while the hyperperfusion in the actual SOZ is not visible due to severe partial volume effects
- a SOZ in the temporal lobe is supported (higher confidence) by hyperperfusion in the ipsilateral insula and/or striatum and/or thalamus and/or motor cortex (seizure propagation from the temporal lobe)
- a SOZ in the temporal lobe is supported (higher confidence) by hypoperfusion in the frontal lobe and/or parietal lobe and/or occipital lobe and/or precuneus (ictal surround inhibition). Regional hypoperfusion on ictal SPECT is often bilateral and does not provide reliable information for the lateralization of the SOZ
- ranking of the confidence regarding the identification of the SOZ: visual identification of the SOZ in the uptake image and significant cluster of hyperperfusion in the statistical parametric map > visual identification in the uptake image only or significant cluster of hyperperfusion in the statistical parametric map only

## **Interictal SPECT**

- the SOZ is identified by regional *hypo*perfusion in cortical grey matter according to visual inspection of the uptake image and/or the statistical parametric map of interictal *hypo*perfusion
- the other way round is not true, that is, regional hypoperfusion in cortical grey matter is not automatically to be interpreted as the SOZ. For example, mild hypoperfusion in the parietal lobe or in the medial frontal cortex (including the anterior cingulate cortex) is rather unspecific and, therefore, not necessarily indicates the SOZ. However, mild hypoperfusion in the parietal lobe or in the medial frontal cortex can indicate the SOZ. Candidates for the SOZ in these brain regions are often rather uncertain (-> low confidence score)
- the SOZ might be identified in the uptake image only, that is, it is not required that the SOZ is confirmed by a cluster of significant hypoperfusion in the statistical parametric map (the conservative threshold of 3.0 z-score points is expected to result in some false negative findings). In particular, the SOZ might be identified by considerable left-right asymmetry in the uptake image. For example, a SOZ in the left temporal lobe might be identified by lower tracer uptake in the left temporal lobe compared to the right temporal lobe, although the (relative) decrease of the interictal tracer uptake in the left temporal lobe does not reach the level of statistical significance and, therefore, is not confirmed by a significant cluster in the statistical parametric map
- identification of the SOZ might also be based primarily on a cluster of significant hypoperfusion in the statistical parametric map, if on visual inspection of the uptake image the regional hypoperfusion does not appear very prominent (the conservative threshold of 3.0 z-score points is expected to result in a rather low rate of false positive findings)
- a statistically significant cluster of regional hypoperfusion located in white matter should not be interpreted as SOZ. Possible explanations include white matter lesions and pronounced partial volume effects secondary to enlarged ventricles
- a statistically significant cluster of regional hypoperfusion located in white matter can extend into cortical grey matter due to partial volume effects caused by the limited spatial resolution of SPECT. This does not indicate hypoperfusion in cortical grey matter and, therefore, should not be interpreted as SOZ
- regional hypoperfusion in the cerebellum should not be interpreted as SOZ
- the interictal perfusion pattern in the cerebellum does not provide reliable information to support lateralization or localization of SOZ candidates in the cerebrum. For example, hypoperfusion in the cerebellar hemisphere contralateral to a cortical defect or lesion might be due to crossed cerebellar diaschisis (independent of epileptic activity) and, therefore, does not increase the likelihood of the defect / lesion to be the SOZ
- apparent reduction of regional perfusion caused by pronounced partial volume / recovery effects due to atrophy / sulcus widening (according to MRI), particularly widening of the Sylvian or longitudinal fissure, should not be interpreted as SOZ
- a perfusion defect caused by a structural defect (according to MRI), secondary to surgical resection or stroke or a cyst or any other structural defect, should not be interpreted as SOZ
- regional hypoperfusion in cortical grey matter in the border zone of a defect might indicate the SOZ if the hypoperfusion appears more widespread and/or more severe than expected from partial volume effects. This is difficult to evaluate and, therefore, often is associated with reduced confidence of a SOZ in the border zone of a defect
- regional hypoperfusion in or close to a structural lesion visible in MRI (e.g., tumor, postencephalitic) might indicate the SOZ. However, it should be taken into account that the lesion might be hypoperfused by itself, that is, independent of epileptic activity. For example, tissue loss due to hippocampal sclerosis is expected to result in mesiotemporal hypoperfusion independent of epileptic activity. Furthermore, regional hypoperfusion close to a hypoperfused structural lesion might be caused by partial volume effects. If regional hypoperfusion in or close to a structural lesion is interpreted as SOZ, putative uncertainty should result in a low confidence score
- the likelihood/confidence that regional hypoperfusion in cortical grey matter in the border zone of a defect (stroke, resection area, cyst …) or in/close to a structural lesion (e.g., tumor, postencephalitic) indicates the SOZ might increase if the interictal perfusion SPECT does not reveal further SOZ candidates
- strongly pronounced regional hypoperfusion not associated with a defect or lesion usually increases the confidence that this region is the SOZ compared to mild hypoperfusion
- ranking of the confidence regarding the identification of the SOZ: visual identification of the SOZ in the uptake image and significant cluster of hypoperfusion in the statistical parametric map > visual identification in the uptake image only or significant cluster of hypoperfusion in the statistical parametric map only

## **Ictal and interictal SPECT side-by-side**

- first assess ictal SPECT alone and interictal SPECT alone, then assess ictal and interictal SPECT side-by-side
- identification of the SOZ can be based on
  - both ictal and interictal SPECT (if ictal SPECT shows hyperperfusion and interictal SPECT shows hypoperfusion in the same brain region)
  - on ictal SPECT alone (if interictal SPECT shows more or less normal perfusion in the “ictal” SOZ)
  - on interictal SPECT alone (if ictal SPECT shows more or less normal perfusion in the “interictal” SOZ)
  - on side-by-side interpretation of ictal and interictal SPECT alone (due to the combination of regional ictal hyperperfusion that is too mild to be interpreted as the SOZ in ictal SPECT alone and regional interictal hypoperfusion in the same brain region that is too mild to be interpreted as the SOZ in the interictal SPECT alone)
- a SOZ candidate from ictal SPECT that shows similar hyperperfusion in interictal SPECT should not be interpreted as SOZ (or with strongly reduced confidence)
- a SOZ candidate from interictal SPECT that shows similar hypoperfusion in ictal SPECT should not be interpreted as SOZ (or with strongly reduced confidence)
- ranking of confidence: SOZ identified in ictal SPECT and in interictal SPECT and in side-by-side interpretation > SOZ candidate in side-by-side interpretation only ≈ SOZ candidate in ictal SPECT only > SOZ candidate in interictal SPECT only
- ictual hyperperfusion in a given brain region combined with interictal hypometabolism in the homologous brain region in the other (contralateral) hemisphere reduces the confidence regarding the lateralization

## **SISCOM**

- first assess ictal SPECT alone, interictal SPECT alone, and ictal and interictal SPECT side-by-side. Then check each resulting SOZ candidate in the SISCOM map. If the SISCOM map confirms the SOZ candidate, it remains a SOZ candidate. If the SISCOM map does not confirm it, the decision should be reconsidered: it might remain a SOZ candidate (with reduced confidence), but more often it will be no longer considered a SOZ candidate
- check for additional SOZ candidates in the SISCOM map. If there are additional candidates, check them by side-by-side interpretation of ictal and interictal SPECT. If one of the exclusion criteria for SOZ candidates from ictal SPECT alone and/or interictal SPECT alone and/or side-by-side interpretation is met, the additional SOZ candidate from the SISCOM map most likely is not the SOZ

## **General considerations**

- the score “no evidence of SOZ” in the first step of the visual interpretation is restricted to cases with (more or less) normal perfusion. The score “no evidence of SOZ” should not be used in cases with 2 (or more) SOZ candidates that all have about the same likelihood to be the actual SOZ. In the latter case, one SOZ candidate should be selected. If the other SOZ candidate is located in the other hemisphere, the confidence regarding the lateralization is low
- in patients with structural defects or lesions, the visual read of the SPECT images should start with careful inspection of the structural lesion / defect and its boundary zone. Then the visual read should continue with the rest of the brain
- localization in the neighborhood of a structural defect or lesion might increase the likelihood of a SOZ candidate to be the true SOZ. That is, if there are several candidates for the SOZ and one of these candidates is close to a structural defect or lesion, this one might be preferred
- the visual read of the rest of the brain might start with the temporal lobes, since the temporal lobe is the pre-scan hypothesis for the localization of the SOZ in the majority (but not all!) of patients referred to perfusion SPECT for presurgical evaluation in most settings. However, the other brain lobes must not be neglected
- higher statistical significance of a regional effect in a statistical parametric map might increase the likelihood of the effect to correctly identify the true SOZ. The statistical significance of an effect is indicated by the brightness of the color (the brighter the more significant). This applies to statistical parametric maps of ictal hyperperfusion, to statistical parametric maps of interictal hypoperfusion, and to SISCOM maps
- in cases with more than one SOZ candidate with similar significance in the statistical parametric map, the SOZ candidate in the temporal lobe (if there is one) might be preferred over the others (this rule is specific for the patient sample of the present study)

1. **Repeat analyses restricted to the experienced reader**

In order to test for a potential impact of the lack of previous experience in reading brain perfusion SPECT of two of the three readers, all analyses reported in the manuscript were repeated for the visual interpretation by the experienced reader alone. For this purpose, the lateralization score and the lateralization confidence score of the experienced reader were combined into an overall “lateralizing” 3-score for the experienced reader: “empty”, “lateralizing with low confidence”, and “lateralizing with high confidence”. A case was considered "empty”, if the experienced reader categorized the case as “no evidence of the SOZ” in the first step of the visual interpretation. A case was considered "lateralizing with low / high confidence” if the experienced reader lateralized the SOZ to the left or to the right hemisphere and scored his confidence regarding the lateralization with ≤ 3 / ≥ 4 of 5 points on the confidence scale.

The proportion of cases that were “lateralizing with high confidence” according to the experienced reader was 15.3 / 46.3 / 54.2% in the “interictal only” / “ictal only” / “full” setting, the proportion of “lateralizing with low confidence” cases was 47.5 / 46.9 / 32.8%, and the proportion of “empty” cases was 37.3 / 6.8 / 13.0% (supplementary figure 3A). The difference of the lateralizing 3-score of the experienced reader in the “interictal only” setting was statistically significant compared to both, the “ictal only” and the “full” setting (both p<0.001). The difference between the “ictal only” and the “full” setting was not significant (p=0.727). The overall lateralization rate (independent of the confidence) by the experienced reader was 62.8 / 93.2 / 87.0% in the “interictal only” / “ictal only” / “full” setting.

The results regarding the comparison of the lateralization performance of the experienced reader between the “interictal only” and the “ictal only” setting with either the “full” setting or favorable surgical outcome as reference standard are summarized in supplementary figures 3C,D and supplementary figures 4, 5, analogous to figures 4C,D and 5, 7 in the manuscript (summarizing the corresponding results for the lateralizing 3-score combining the lateralization scores and the lateralization confidence scores from the 3 independent readers).

There were two obvious differences. First, the proportion of “empty” cases according to the experienced reader was consistently smaller than the proportion of “non-lateralizing” cases according to the combined lateralization 3-score from the 3 independent readers. This was to be expected, given that for a case to be “non-lateralizing” according to the 3 independent readers, it was sufficient that only one of the 3 independent readers categorized the case as “no evidence of the SOZ”. Furthermore, “non-lateralizing” according to the 3 independent readers included also cases with inconsistent lateralization (to different hemispheres) across the 3 readers. Second, the proportion of cases with “high confidence” lateralization was consistently higher for the experienced reader than for the 3 readers combined. This was to be expected, too, given that for a lateralizing case to be of low confidence only according to the 3 independent readers it was sufficient that only one of the 3 readers scored ≤ 3 of 5 confidence points. Furthermore, less experienced raters often tend to score with lower confidence.

However, the relationship of the visual interpretation between the 3 considered settings (“interictal only”, “ictal only”, “full”), both among each other and relative to the favorable surgical outcome as reference, was very similar for the lateralizing 3-score of the experienced reader and for the lateralizing 3-score combining the 3 independent readers. This suggests that the findings of the current study regarding its primary aim, the added value of ictal SPECT after high-confidence lateralization of the SOZ by interictal SPECT and vice versa, are not limited by the varying experience of the readers.

**References to the supplementary material**

1. Dupont P, Zaknun JJ, Maes A, Tepmongkol S, Vasquez S, Bal CS, et al. Dynamic perfusion patterns in temporal lobe epilepsy. Eur J Nucl Med Mol Imaging. 2009;36:823-30. doi:10.1007/s00259-008-1040-6.

2. Shin WC, Hong SB, Tae WS, Seo DW, Kim SE. Ictal hyperperfusion of cerebellum and basal ganglia in temporal lobe epilepsy: SPECT subtraction with MRI coregistration. J Nucl Med. 2001;42:853-8.

**Supplementary Figures**


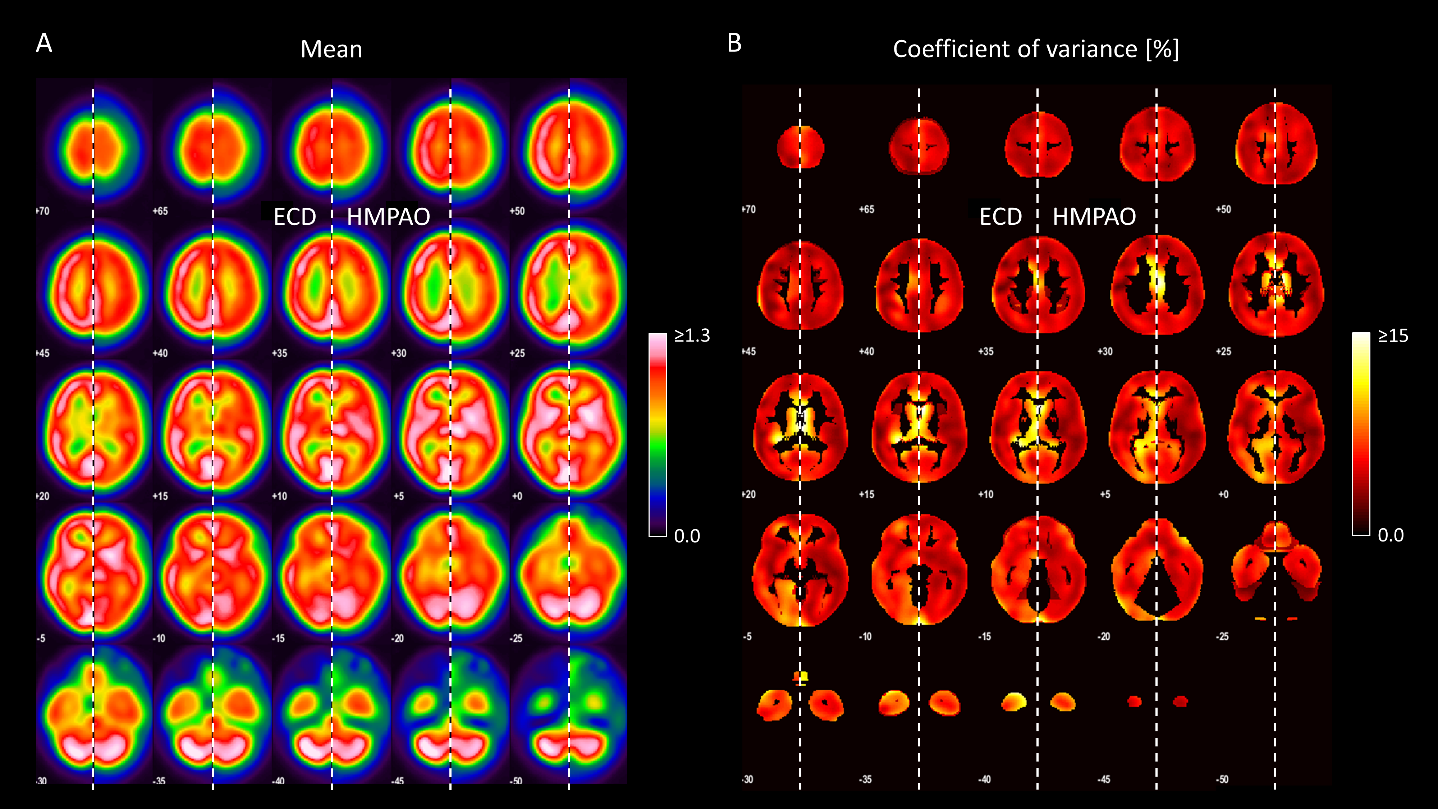


**Supplementary Fig. 1** Voxelwise mean (A) and voxelwise coefficient of variance (B) in the custom ^99m^Tc-ECD normal database and in the custom ^99m^Tc-HMPAO normal database in MNI space. In each slice, the left half presents ^99m^Tc-ECD, the right half presents ^99m^Tc-HMPAO

**
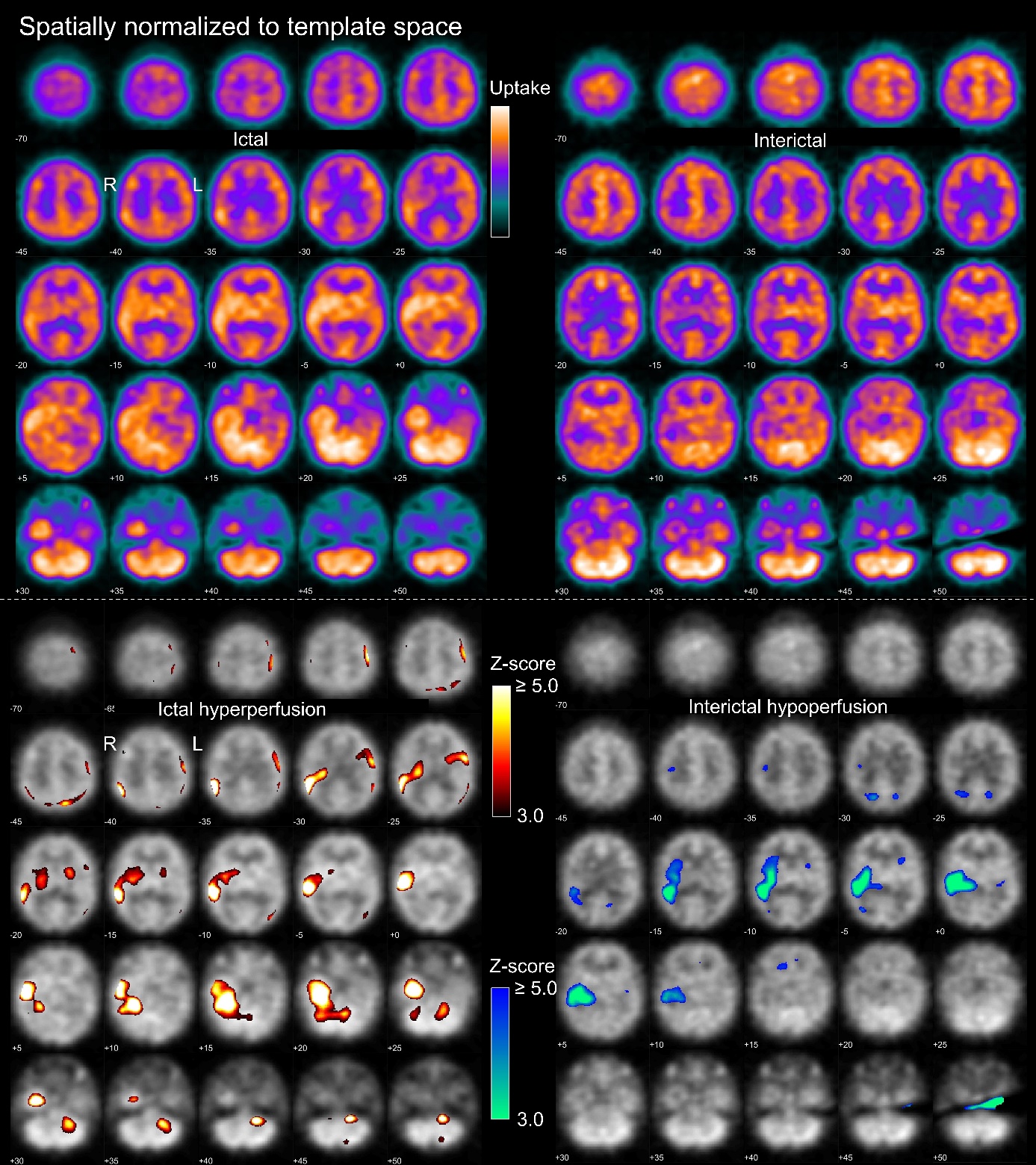
**

**Supplementary Fig. 2** Standardized side-by-side display of ictal and interictal SPECT for visual interpretation in the “full” setting. The upper and lower parts were provided as page 5 and page 6 of an 8-page pdf-document. Page 5 shows ictal and interictal uptake image in MNI space. Page 6 shows the statistical maps of ictal hyperperfusion and interictal hypoperfusion thresholded at z ≥ 3.0 and overlaid to the ictal and interictal uptake image, respectively. The example images are from the same patient as in Figures 1-3 in the manuscript


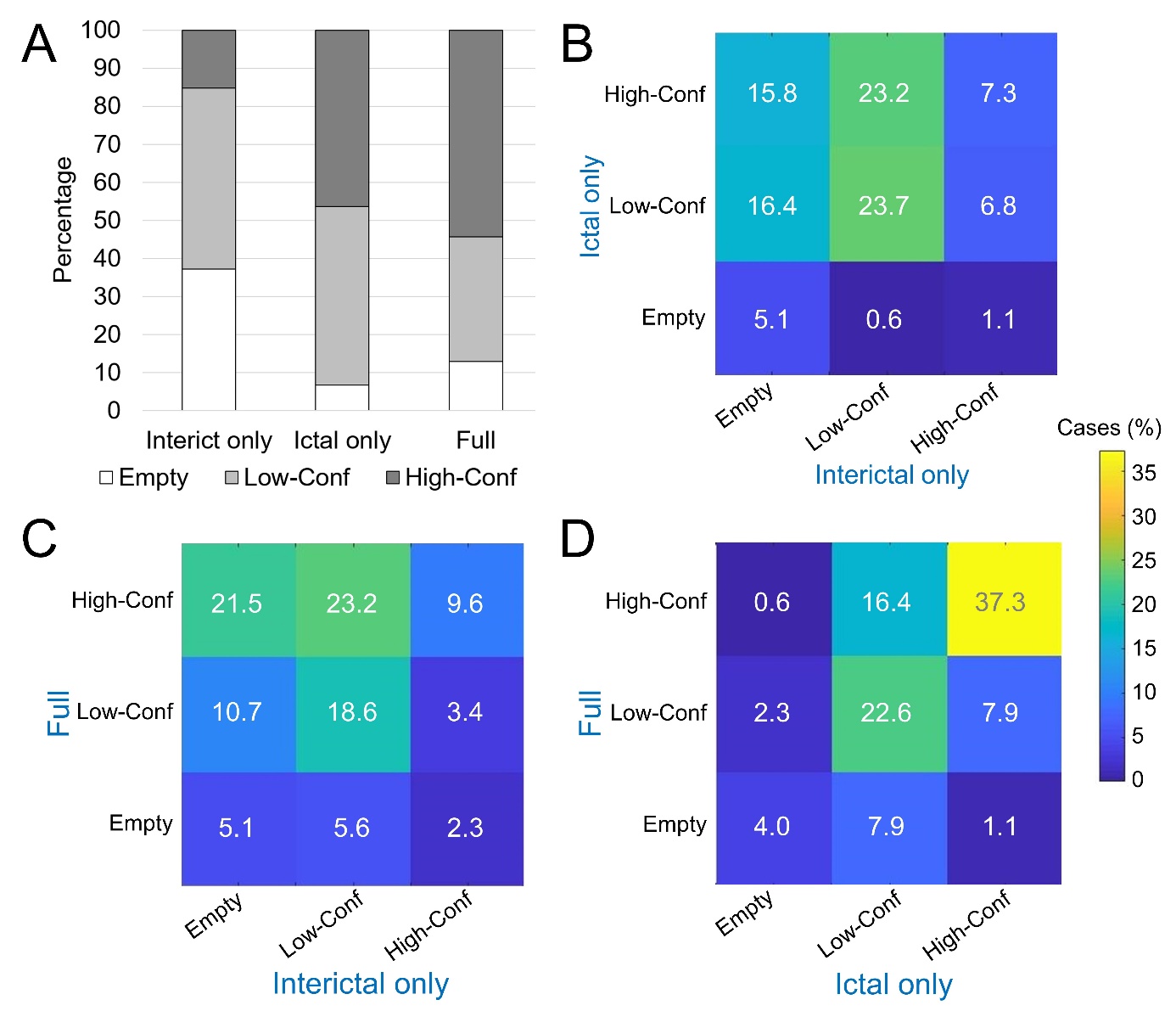


**Supplementary Fig. 3** Between-settings differences of the lateralizing 3-score of the experienced reader. Proportion of “empty”, “lateralizing with low confidence” (Low-Conf) and “lateralizing with high confidence” (High-Conf) cases according to the visual interpretation by the experienced reader in each of the three settings (“interictal only”, “ictal only”, “full”). B-D: Cross-tables of the lateralizing 3-score according to the experienced reader for each pair of settings. The numbers represent the percentages of cases relative to the whole patient sample (n = 177). The corresponding results for the lateralizing 3-score obtained by combining lateralization and confidence scores across the 3 independent readers are shown in Figure 4 in the manuscript.


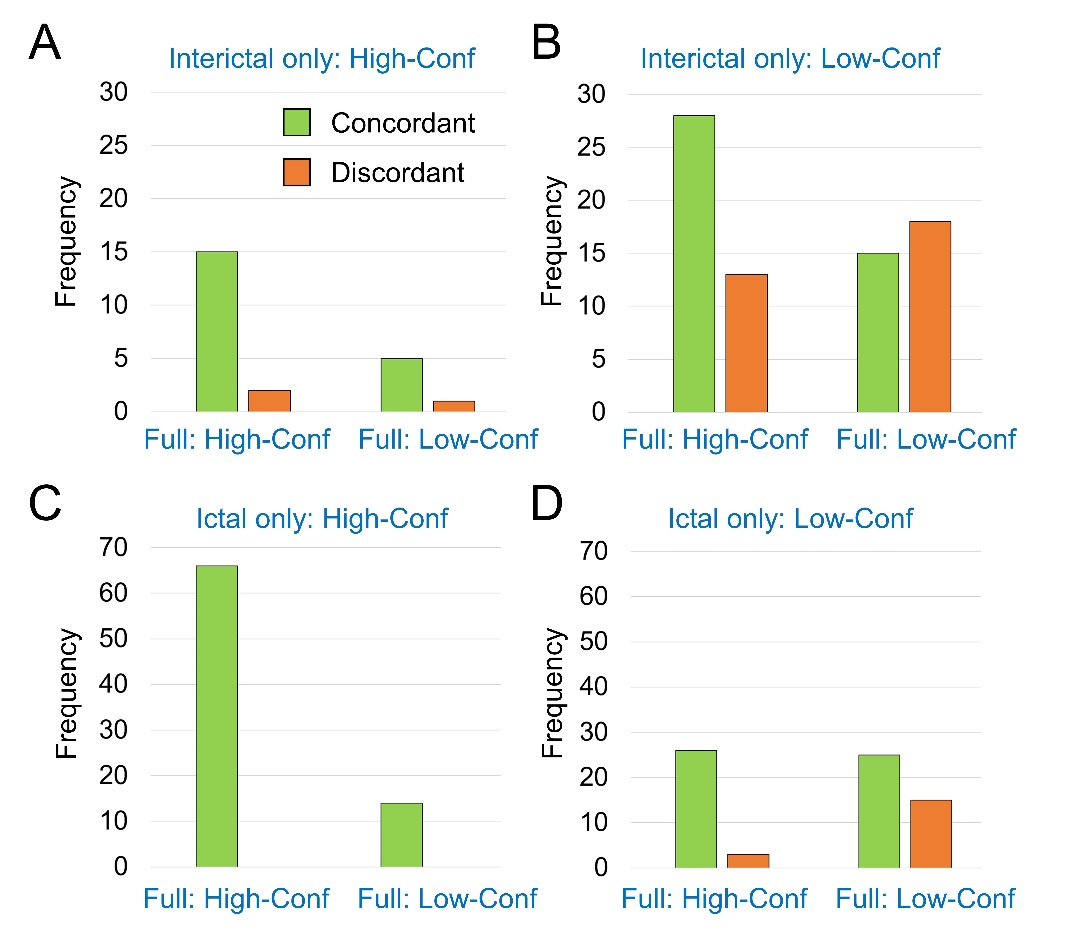


**Supplementary Fig. 4** Concordant and discordant lateralization by the experienced reader relative to the “full” setting. Frequency (number of cases) of concordant lateralization (same hemisphere) and discordant lateralization (different hemispheres) compared to the “full” setting according to the visual interpretation by the experienced reader, separately for the “interictal only” setting (A, B) and the “ictal only” setting (C, D), and separately for “lateralization with high confidence” (A, C) and “lateralization with low confidence” (B, D). In each of the subplots (A-D), the cases are sorted according to the confidence regarding the lateralization in the “full” setting (left: high confidence, High-Conf; right: low confidence, Low-Conf). Cases that were “empty” in the “full” setting are excluded (Figure R2 C, D). The scaling of the vertical axis differs between the “interictal only” setting (A, B: 0-30) and the “ictal only” setting (C, D: 0-70) to account for the lower number of lateralizing cases in the “interictal only” setting. The corresponding results for the lateralizing 3-score obtained by combining lateralization and confidence scores across the 3 independent readers are shown in Figure 5 in the manuscript.

**
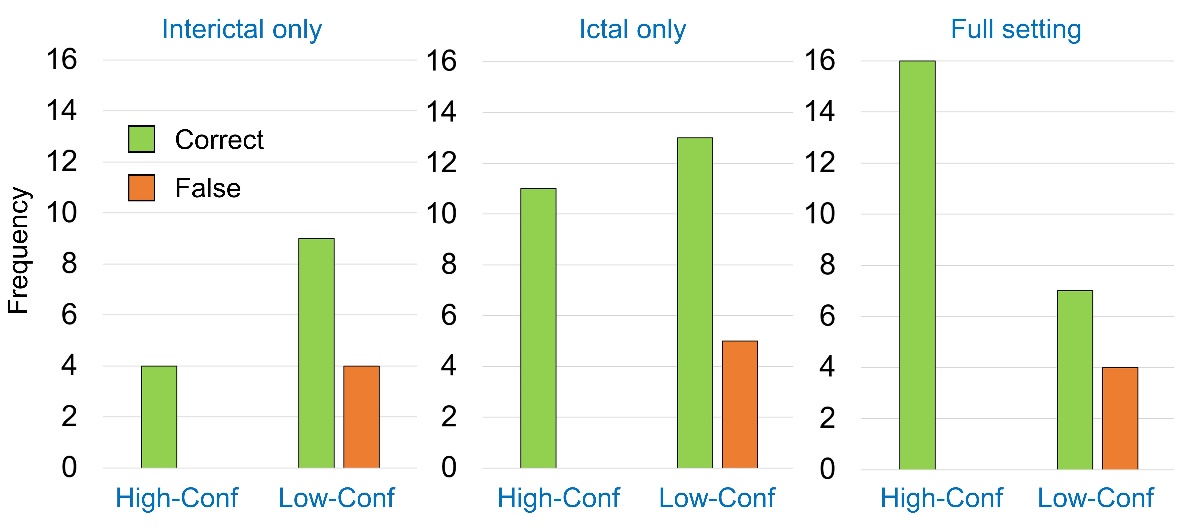
**

**Supplementary Fig. 5** Lateralization accuracy of the experienced reader relative to favorable surgical outcome. Frequency (number of cases) of correct lateralization (to the operated hemisphere, green) and false lateralization (contralateral to the operated hemisphere, orange) by the experienced reader in 31 patients with favorable seizure outcome (Engel I or II) 12 months after temporal lobe surgery. The frequencies are shown separately for each of the 3 considered settings (“interictal only”, “ictal only”, “full” setting) and separately for “lateralization with high confidence” and “lateralization with low confidence” (left: high confidence, High-Conf; right: low confidence, Low-Conf). Non-lateralizing cases were excluded, separately for each setting. The corresponding results for the lateralizing 3-score obtained by combining lateralization and confidence scores across the 3 independent readers are shown in Figure 7 in the manuscript.
